# A germ cell tumor in a patient with swyer syndrome with ambiguous genitalia

**DOI:** 10.1186/s13104-015-1688-5

**Published:** 2015-12-07

**Authors:** Dharshana Tharanga Kulathilake, Champa Jayasundara

**Affiliations:** Sri Jayewardenepura General Hospital, Sri Jayawardenepura Kotte, Sri Lanka

**Keywords:** Dysgerminoma, Swyer syndrome, 46 XY complete gonadal dysgenesis, Disorders of sex development

## Abstract

**Background:**

Swyer syndrome is a rare manifestation of disorders of sex development in which the individual is 46, XY in genotype but phenotypically a female. They have normal female external genitalia with under developed female internal genitalia. They usually present with primary amenorrhoea and delayed puberty but also can present with gonadal tumors in adult life.

**Case presentation:**

A 25-year-old Sri Lankan phenotypically female having 46 XY karyotype with a history of primary amenorrhoea, presented with left loin pain associated with fever. General examination revealed a tall stature, scanty axillary and pubic hair, small breasts and clitoromegally. A tender ill-defined mass was detected in the left hypochondrial region. She had high erythrocyte sedimentation rate with elevated alkaline phosphatase and lactate dehydrogenase levels. Her serum hormonal assay revealed a low estradiol level with elevated luteinizing hormone and follicular stimulating hormone levels with normal progesterone and testosterone levels. Computerized tomography of abdomen showed a large complex mass lesion in relation to antero-medial aspect of the lower pole of the left kidney with para-aortic and left common iliac lymph nodes. The diagnostic laparoscopy confirmed the presence of internal female genitalia and the mass lesion was seen in left para-aortic region. The histology revealed a germ cell tumor compatible with a dysgerminoma.

**Conclusion:**

Patients with Swyer syndrome can present with gonadal tumors, typically a dysgerminoma in their adult life.

## Background

Swyer syndrome or 46, XY complete gonadal dysgenesis (46, XY CGD) is a disorder of sex development (DSD) in which the individual is 46, XY in genotype but phenotypically a female. They have unambiguous female external genitalia and normal Müllerian structures. Mutations in the genes SRY, DAX1, WNT4, SOX9, SF1, WT1 and WMRT1—WMRT2 in chromosome Y have been considered responsible for the development of 46, XY CGD. Patients with Swyer syndrome show hypergonadotrophic hypogonadism with low levels of estrogens and normal female levels of androgens and they usually present with primary amenorrhoea and delayed puberty. The syndrome may also present in late adulthood with gonadal tumors, typically a dysgerminoma. We report a case of a 23-year-old phenotypic female with 46, XY genotype and female internal genitalia, ambiguous external genitalia (clitoromegally) presenting with a germ cell tumor in an unusual site, considered as a dysgerminoma in a case of Swyer’s syndrome.

## Case presentation

A 25-year-old Sri Lankan phenotypically female presented to the general medical unit with the complaint of gradual onset left loin pain associated with fever for 5 days. It was a moderate to severe dull ache which did not radiate to groin. The fever was intermittent and associated with chills but not with rigors. She did not experience urinary symptoms like dysuria, increased urinary frequency or hematuria. She has had few episodes of vomiting associated with the abdominal pain but her bowel opening was normal. She’s an unmarried, nulliparous female and further inquiry revealed that she had been investigated for primary amenorrhoea at the age of 14 years. She was found to have rudimentary uterus with intact ovaries ultrasonically and was initially started on combined oral contraceptive pills. With the medication she started normal menstruation but the treatment was not regularly taken due to the appearance of acne and weight gain. At the age of 23 years she was referred to an Endocrinologist and found to have 46, XY genotype by chromosomal analysis. She was given estradiol valerate titrating doses by the physician but treatment was defaulted by the patient due to fear of cancer risk.

On examination she was 66 kg in weight and 170.2 cm in height (BMI = 22.84 kg/m^2^). She had small breasts, scanty axillary hair and scanty female pattern pubic hair. Her external genitalia were female in type but clitoromegally was observed. She was febrile but not pale or icteric. Cervical lymphadenopathy was not detected. A tender mass with smooth surface was palpated over left hypochondrium which was not ballotable and the upper margin was not detected by palpation or by percussion. There was no hepatomegaly. Her cardiovascular, respiratory neurological and loco-motor system examinations were unremarkable.

**Fig. 1:**
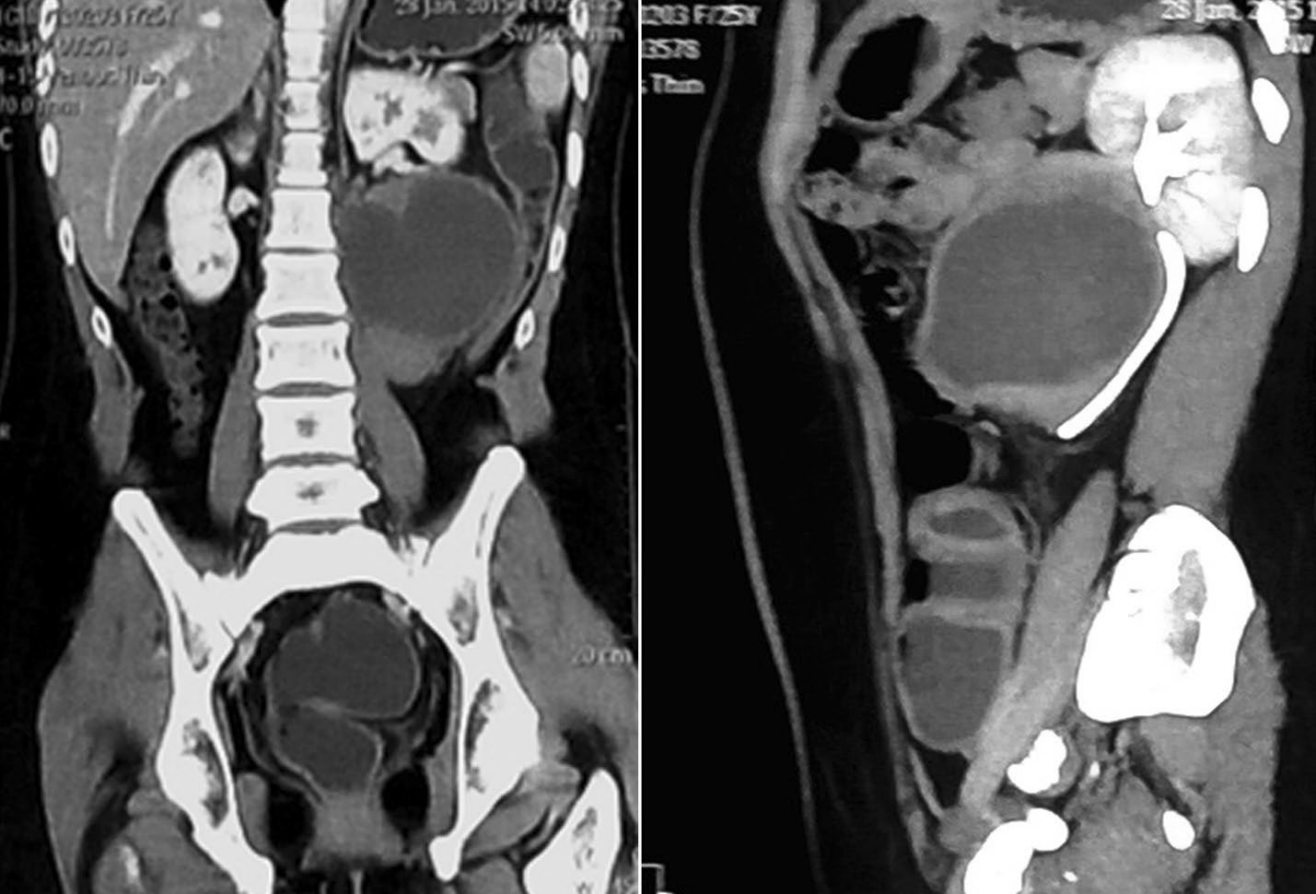
Contrast enhanced computerized tomographic image of the tumor. Contrast enhanced computerized tomography of abdomen and pelvis showing large tumor in relation to the lower pole of the left kidney

Investigations revealed a total white cell count of 7.6 × 10^9^/ml (neutrophils—75.3 %, lymphocytes—13.9 %, monocytes—8.3 % and eosinophils—2.4 %), hemoglobin of 11.5 g/dl and a platelet count of 243 × 10^9^/ml. Erythrocyte sedimentation rate was 127 mm in 1st hour. Liver profile revealed alkaline phosphatase (ALP) level of 2986 U/L with slightly elevated transaminase levels (alanineaminotransferase—60 U/L and aspartate aminotransferase—47 U/L). Renal and coagulation profiles were normal. Serum lactate dehydrogenase (LDH) level was 395 U/L. Serum tumor markers including alpha-feto protein (AFP) levels, serum beta- human chorionic gonadotropin (hCG) and CA 125 levels were within normal range. The chest and lumbo-sacral spine roentgenograms were reported as normal. Ultrasound scan of abdomen and pelvis revealed a well-defined mass lesion in relation to the lower pole of the left kidney. Small uterus was visualized but ovaries could not be identified separately. The liver was normal in size and there were no focal lesions. Contrast enhanced computerized tomography of abdomen and pelvis revealed large complex mass lesion (11.0 × 9.7 × 8.3 cm) in relation to antero-medial aspect of the lower pole of the left kidney (Fig. 1). Few para-aortic and left common iliac lymph nodes were also detected.

Her serum hormonal assay revealed a low estradiol level of 9.0 pg/ml (normal range: 39–189 pg/ml in follicular phase) in the background of elevated luteinizing hormone level of 40.64 μIU/ml (2–15 μIU/ml in follicular phase) and follicular stimulating hormone level of 73.09 μIU/ml (3–20 μIU/ml in follicular phase). The progesterone level of 0.35 ng/ml (0.33–1.2 ng/ml in follicular phase) and testosterone level of 0.25 ng/ml (0–0.73 ng/ml) were within normal range. She was found to have normal thyroid stimulating hormone level of 2.55 μIU/ml (0.4–5.1 μIU/ml) and serum prolactin level of 18.5 ng/ml (3.9–29.5 ng/ml).

**Fig. 2:**
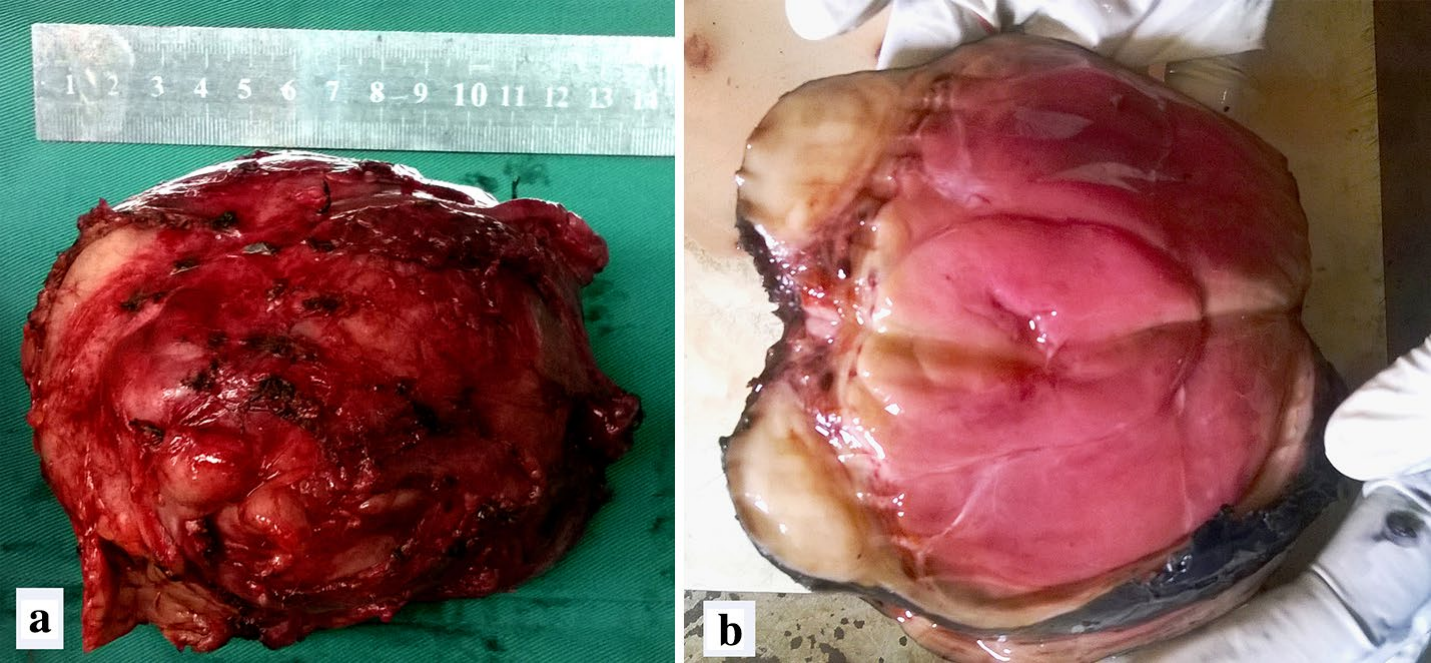
Macroscopic view of the tumor. A well encapsulated vaguely lobulated mass with a smooth surface measuring 11.0 cm × 10.5 cm × 9.0 cm (**a**). Cut section showing a uniform *red-brown* necrotic mass with a firm, *yellow-white* separate lobule outside the capsule measuring 4.0 cm × 0.0 cm × 2.8 cm (**b**)

**Fig. 3:**
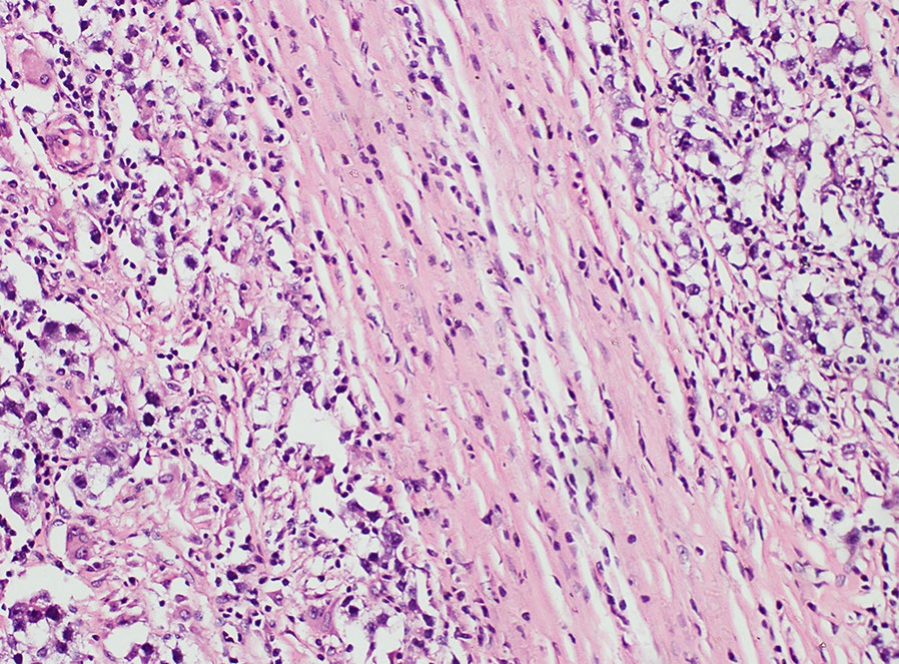
Microscopic view of the tumor. Microscopy of the *yellow-white* lobule showing large vesicular cells with clear cytoplasm well defined cell boundaries and centrally placed regular nuclei. The stroma is infiltrated with lymphocytes

Diagnostic laparoscopy revealed a small uterus with bilateral normal sized ovaries with fallopian tubes. The mass lesion revealed by imaginary studies was confirmed as a para-aortic mass but which was not attached to the lower pole of the left kidney. The left gonadal vein was seen crossing over the mass. Excision of the left para-aortic mass with attached lymph nodes was done via a subcostal incision which extended up to the xyphisternum. Pathological sectioning of the mass macroscopically showed a soft degenerating tumor with a uniform red-brown appearance (Fig. 2a). A uniform, firm, yellow- white separate lobule was seen outside the capsule measuring 4.0 × 3.0 × 2.8 cm (Fig. 2b). Five lymph nodes were dissected from the loose connective tissue outside the capsule. Sections of large red brown mass showed a necrotic mass of tumor with few areas of viable tissue. Adjacent white lobule showed viable tumor tissue compatible with germ cell tumor consistent with a seminoma or dysgerminoma (Fig. 3). Tumor nodules were noted within the capsule and were seen to infiltrate beyond the capsule into adjacent connective tissue. No vascular or perineural invasion was noted. Normal testicular or ovarian tissue was not detected within the tumor mass. Four out of five lymph nodes showed tumor deposits within which two nodes were extensively involved by the tumor.

The differential diagnosis of Swyer syndrome (46, XY complete gonadal dysgenesis), 46, XY disorder of sex development (46, XY DSD) and partial androgen insensitivity syndrome were made. Androgen insensitivity syndrome was easily excluded since the presence of female internal genital organs. Even though the presence of clitoromegally is against the diagnosis of Swyer syndrome, 46, XY DSD could be fairly excluded due to the absence of testicular elements via direct visualization of the abdominal cavity and by histology. The treating oncologist decided to preserve the ovaries and the patient was given full course of chemotherapy.

## Discussion

Swyer syndrome or 46, XY CGD is a disorder of sex development (DSD) in which the individual is 46, XY in genotype but phenotypically a female with unambiguous female external genitalia and normal Müllerian structures. These patients’ mesonephric ducts (Wolffian ducts) get atrophied while paramesonephric ducts (Müllerian ducts) develop into uterus, fallopian tubes and vagina in the absence of testosterone and anti-Müllerian hormone. However, such female patients, in the absence of XX chromosome do not have a properly developed uterus and ovaries.

Mutations in the SRY gene in chromosome Y have been implicated as the basic etiology, but duplication of DAX1 (also known as NROB1) and WNT4 genes as well as haplo-insufficiency of the SOX9, SF1, WT1 and WMRT1—WMRT2 genes are also have been considered responsible for the development of 46, XY CGD [1]. SRY gene plays a pivotal role in human testis development and its inactivation mutations will lead to complex XY sex reversal as seen in 46, XY CGD. But the mutations in the SRY gene are found only in 10–20 % of cases of 46, XY CGD [2]. The majority are located within the HMG box, a DNA binding domain which acts as a modulating local chromatin structure in order to transcribe adjacent target genes. The majority of mutations are de novo, yet there is a subset of cases where the mutation runs in families, but index cases are been borne to phenotypically normal and fertile fathers. One explanation for these familial cases is paternal gonadal mosaicism [3]. The incidence of Swyer syndrome is 1:100,000 [4].

Patients with Swyer syndrome show hypergonadortrophic hypogonadism with low levels of estrogens and normal female levels of androgens. Their minimal breast development is a result of peripheral aromatization of androgens. They have scanty pubic and axillary hair and are taller than the ordinary females. Their gonads (ovaries) display fibrous tissue but no follicles [5].

They usually present with primary amenorrhoea and delayed puberty. The syndrome may also present in late adulthood with gonadal tumors, typically a dysgerminoma. Other tumors with which they can manifest are gonadoblastoma, teratoma and embryonic carcinoma. The lifetime risk of gonadal tumors is in range of 15–35 % [3].

Since the Müllerian structures are preserved in Swyer syndrome the uterus may increase in size when estrogen replacement is started and successful pregnancies have been achieved following egg donation.

46, XY partial gonadal dysgenesis, which is now called as 46, XY disorder of sex development (46, XY DSD) is also a DSD associated with anomalies in gonadal development that results in genital ambiguity of variable degree ranging from clitoromegally in female phenotype to absolute male phenotype in a patient with 46, XY karyotype. It is associated with abnormality of both Leidig and Sertoli cell function that may result from deletions or point mutations in the SRY gene or dose sensitive (RROB1) locus duplication on the X chromosome [6]. These patients have dysgenetic gonads with testicular elements with reduced to absent sperm production. Müllerian structures including uterus and fallopian tubes may or may not be present in these cases.

Ovarian germ cell tumors are derived from primordial germ cell tumors of the ovary and can be benign or malignant. Dysgerminomas are the female version of the male seminoma and are essentially composed of immature germ cells. They can occur at any age but have a predilection for young women. Grossly, dysgerminoma appears as a lobulated mass that is firm and cream colored or pale tan. Histologically, the neoplasm is composed of undifferentiated germ cells, large vesicular cells with clear cytoplasms, well defined cell boundaries and centrally placed regular nuclei. The overall picture is also described as “fried-eggs” appearance. The stroma is infiltrated by clusters of small lymphocytes and frequently contains granulomas. Due to the rapid growth of dysgerminoma, patients usually present with abdominal enlargement and pain due to rupture with hemoperitoneum or torsion. Dysgerminomas can contain syncytiotrophoblastic giant cells those produce placental ALP and LDH. In addition, 3–5 % of dysgerminomas produce hCG, but in general, they do not produce AFP [7]. Surgery is performed for definitive diagnosis, staging and initial treatment of dysgerminomas.

Dysgerminomas may develop within a gonadoblastoma (a benign or in situ germ cell ovarian neoplasm composed of germ cells and sex cord stroma) in phenotypic females who have a Y chromosome. This group includes patients with 46, XY complete gonadal dysgenesis, 46, XY disorder of sex development and complete androgen insensitivity.

## Conclusion

We report a possible case of Swyer syndrome with ambiguous genitalia, who was diagnosed as having 46 XY genotype at the age of 23 years and lost to follow up for the next 2 years presenting with advanced (stage IIIC) germ cell tumor emphasizing the need of close monitoring of these type of patients by a multidisciplinary team. The clinical picture fits to the 46, XY CGD, but opens this presentation to a new entity in the classification of disorders of sex development, since linked with ambiguous genitalia and should be further evaluated.

## Consent

Written informed consent was obtained from the patient for publication of this case report and any accompanying images.

## Abbreviations

CGD: complete gonadal dysgenesis
DSD: disorder of sex development
ALP: alkaline phosphatase
LDH: lactate dehydrogenase
AFP: alpha-feto protein
hCG: human chorionic gonadotropin

## References

[1] Xue DU, Xuhong Zhang, Yongmeli Li, Yukun Han (2014). 46, XY female sex reversal syndrome with bilateral gonadoblastoma and dysgerminoma. Exp Ther Med.

[2] Thomas FG, King, Conway Gerard S (2014). Swyer syndrome: a review. *Reproductive*. Endocrinology.

[3] Hughes IA, Warrell David A, Cox Timothy M, Firth John D (2010). Normal and abnormal sexual differentiation. Oxford textbook of medicine.

[4] Fernandes Gwendolyn C, Sathe Pragati A, Nalik Leena P, Kane Shubhada V (2010). Bilateral gonadoblastoma with unilateral dysgerminoma in a case of 46, XY pure gonadal dysgenesis (Swyer syndrome). Indian J Pathol Microbiol.

[5] Han (2011). Dysgerminoma in a case of 46, XY pure gonadal dysgenesis (Swyer syndrome): a case report. Diagn Pathol.

[6] Ostrer H. 46, XY Disorder of sex development and 46, XY complete gonadal dysgenesis. In: Pagon RA, Adam MP, Ardinger HH et al., editors. GeneReviews^®^ [Internet]. Seattle: University of Washington; 2008. pp 1993–2015. http://www.ncbi.nlm.nih.gov/books/NBK1547/. **(Updated 15 Sep 2009)**.

[7] Gershenson DM. Ovarian germ cell neoplasms: pathology, clinical manifestations, and diagnosis. In: UptoDate (Version 18).

